# Selective inhibition of LY294002 across cloned human cardiac ion channels: implications for atrium-selective therapy in atrial fibrillation

**DOI:** 10.3389/fphar.2026.1899932

**Published:** 2026-09-09

**Authors:** Rui Niu, Jiawei Li, Huanle Fang, Wei Zhao, Chao Dong, Ningjuan Han, Mengyuan Chen, Lie Ma, Liang Li, Yun Fan, Bing Zhu, Beibei Bie, Jie Wu, Hiroshi Matsuura, Minoru Horie

**Affiliations:** 1 Medical School of Xi’an Peihua University, Xi’an, Shaanxi, China; 2 Guangdong-Hong Kong-Macao Greater Bay Area Center for Drug Evaluation and Inspection, National Medical Products Administration, Shenzhen, Guangdong Province, China; 3 Department of Pharmacy, The First Affiliated Hospital of Xi’an Medical University, Xi’an, China; 4 Innovation Laboratory for Natural Medicine Research and Translation, Xi’an Peihua University, Xi’an, China; 5 Department of Physiology, Shiga University of Medical Science, Otsu, Shiga, Japan; 6 Department of Cardiovascular and Respiratory Medicine, Shiga University of Medical Science, Otsu, Shiga, Japan

**Keywords:** atrial fibrillation, cardiac ion channels, CHO cells, LY294002, patch-clamp technique

## Abstract

**Background and purpose:**

Atrial fibrillation (AF) is the most prevalent clinical arrhythmia, yet current antiarrhythmic pharmacotherapies have limited efficacy and pose risks of off-target ventricular block. LY294002 was reported to inhibit the ultrarapid delayed-rectifier potassium current (*I*_Kur_) carried by Kv1.5 channels, a promising atrium-selective therapeutic target for AF. To determine whether LY294002 exhibits functional selectivity for *I*_Kur_, this study comprehensively examined its inhibitory effects on major counter-target cardiac ion currents.

**Methods:**

The cDNAs encoding the principal pore-forming subunits of major cardiac channels—mediating *I*_Kr_(Kv11.1), *I*_Ks_(Kv7.1), *I*_K1_(Kir2.1), *I*_to_(Kv4.3), *I*_Na_(Nav1.5), and *I*_Ca-L_(Cav1.2)—were heterologously expressed in Chinese hamster ovary cells. The inhibitory effects of LY294002 on these channels were assessed using the whole-cell patch-clamp technique. Furthermore, molecular docking analysis was conducted to characterize the structural interactions and binding configurations of LY294002 with Kv1.5.

**Results:**

LY294002 inhibited all investigated currents in a concentration-dependent manner, albeit with markedly varying potencies. Based on Hill equation fits, the half-maximal inhibitory concentration (IC_50_) values for LY294002 on *I*_Ks_, *I*_Kr_ *, I*_to_ *, I*_K1_, *I*_Na_, and *I*_Ca-L_ were 21.5, 26.3, 55.9, 81.5, 131.6, and 46.5 μM, respectively. Notably, the lowest off-target IC_50_ (*I*_Ks_) was approximately 2.7-fold higher than the previously reported IC_50_ for *I*_Kur_ (7.9 μM). Molecular docking analysis revealed that LY294002 bound within the internal cavity of Kv1.5, with predicted binding affinity of −8.3 kcal*I*mol^−1^.

**Conclusion:**

The inhibitory potency of LY294002 against *I*_Kur_ is substantially higher than its potency against other major cardiac currents, demonstrating its potential to act as a selective *I*_Kur_ blocker. Structurally, this preferential inhibition is likely mediated by the compound’s favorable occupancy of key gating or pore regions unique to the Kv1.5 channel conformation.

## 1 Introduction

Atrial fibrillation (AF) is the most prevalent sustained cardiac arrhythmia and a major risk factor for stroke, heart failure, and overall mortality, with incidence rising sharply with age (Ko et al., 2025). In 2020, the global burden of AF encompassed an estimated 50 million individuals (Joglar et al., 2024). However, currently available antiarrhythmic drugs for AF suffer from limited efficacy and carry substantial risks of severe adverse effects, most notably life-threatening ventricular proarrhythmias (Heijman et al., 2021). These limitations have driven ongoing efforts to develop safer rhythm-control agents with superior cardiac chamber selectivity and well-defined mechanisms of action (Saljic et al., 2022).

A promising therapeutic strategy for treating cardiac arrhythmias involves targeting specific ion channels to minimize off-target ventricular side effects. The Kv1.5 channel, encoded by *KCNA5*, mediates the ultrarapid delayed-rectifier potassium current (*I*_Kur_), which is predominantly expressed in human atria but functionally negligible in the ventricles. Selective inhibition of Kv1.5 is considered an effective approach to suppress atrial reentrant and triggered activities by prolonging the atrial action potential duration and effective refractory period, without risking excessive ventricular repolarization prolongation (Dong et al., 2022). Nevertheless, because other concomitant cardiac currents—such as the inward Na^+^ current (*I*_Na_), L-type calcium current (*I*_Ca-L_), transient outward potassium current (*I*_to_), inward rectifier potassium current (*I*_K1_), slow delayed-rectifier potassium current (*I*_Ks_), and rapid delayed-rectifier potassium current (*I*_Kr_)—profoundly influence cardiac excitability, refractoriness, and rhythm, unintended alterations in these channels can severely disrupt cardiac function (Camargo-Ayala et al., 2025). Therefore, evaluating selective action on Kv1.5/*I*_Kur_ relative to these major ventricular currents is crucial for developing safe, atrium-selective antiarrhythmic agents.

**FIGURE 1:**
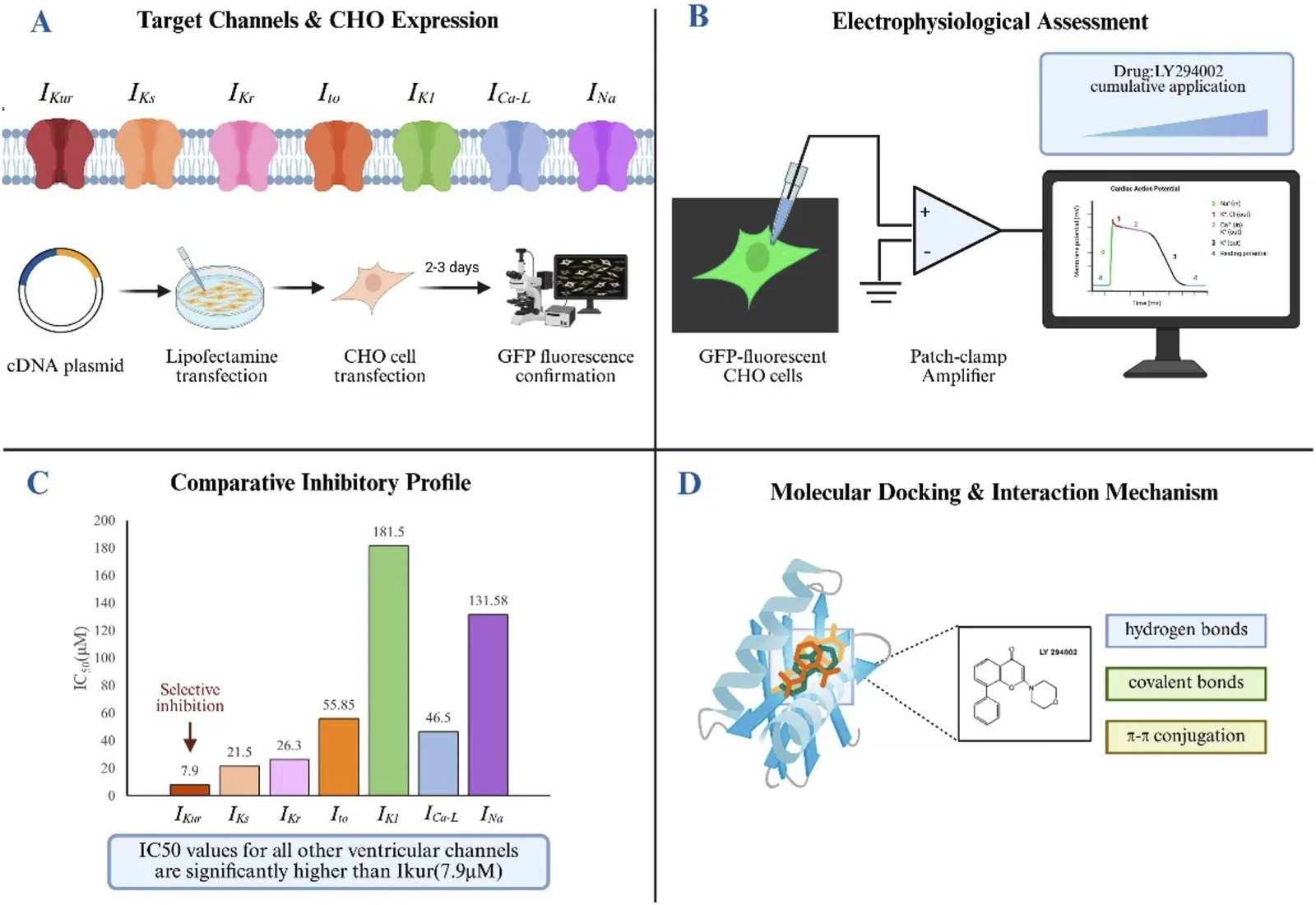
Workflow for assessing the selective inhibition of LY294002 on *I*_Kur_. **(A)** Schematic overview of the experimental workflow: a panel of major cardiac ion channels is depicted; CHO cells heterologously expressing Kv1.5 were established and cultured for subsequent whole-cell patch-clamp recordings. **(B)** Whole-cell patch-clamp recording and dosing paradigm: LY294002 was applied using a cumulative application protocol, and current changes over time/concentration were recorded to generate inhibition profiles. **(C)** Bar plot comparing IC_50_ values across ion channels: LY294002 showed the lowest IC_50_ toward *I*_Kur_ (Kv1.5), whereas markedly higher IC_50_ values were observed for other cardiac ion channels, indicating a relatively selective inhibitory effect on *I*_Kur_. **(D)** Molecular docking: LY294002 adopted a stable binding pose within the channel pocket with favorable predicted binding energy and was predicted to engage in hydrogen bonding, hydrophobic/covalent contacts, and π–π conjugation interactions, providing a molecular rationale for its inhibitory activity.

LY294002, a specific phosphoinositide 3-kinase (PI3K) inhibitor, is chemically derived from the naturally occurring bioflavonoid quercetin. Our previous work demonstrated that LY294002 acts as an open-channel blocker to inhibit cloned human Kv1.5 (hKv1.5) channels heterologously expressed in Chinese hamster ovary (CHO) cells, operating in a concentration-dependent but PI3K-independent manner (Wu et al., 2009). In the present study, we utilized the whole-cell patch-clamp technique combined with molecular docking analysis to comprehensively investigate the effects of LY294002 on six major cardiac ion channels. Our primary objective was to elucidate the mechanism underlying the selective inhibition of LY294002 on the Kv1.5/*I*_Kur_ system, thereby providing a structural and functional reference for the development of novel, atrium-selective antiarrhythmic agents for the management of AF. The overall experimental design and research workflow of this study is illustrated in Figure 1.

## 2 Materials and methods

### 2.1 Heterologous expression of cardiac ion channels

Full-length cDNA encoding human *KCNQ1*, subcloned into a pIRES2-EGFP expression vector containing enhanced green fluorescent protein (GFP), was a kind gift from Dr. J. Barhanin (Institut de Pharmacologie Moleculaire et Cellulaire, CNRS, Valbonne, France). Full-length cDNA encoding human *KCNE1* (GenBank M26685), subcloned into a pcDNA3.1 expression vector, was obtained by PCR from a human heart cDNA library (Clontech Laboratories, Mountain View, CA). Full-length cDNA encoding human *KCNH2* (*hERG*), subcloned into a pRc/CMV expression vector, was a kind gift from Dr. M. Sanguinetti (University of Utah, Salt Lake City, UT, United States). Full-length cDNA encoding the short isoform of human Kv4.3 (*KCND3*), subcloned into the pIRES-GFP expression vector, was kindly provided by Dr. G.F. Tomaselli (Johns Hopkins University, MD, United States). Full-length cDNA encoding human *KCNIP2*, subcloned into a pCMV-IRS expression vector, was a kind gift from Dr. G.-N. Tseng (Virginia Commonwealth University, Richmond, VA, United States). Full-length cDNA encoding a *KCNE2* fragment, originally carried in a pCR3.1 vector, was subcloned into a pIRES-CD8 expression vector. Full-length cDNA encoding human *KCNJ2*, subcloned into a pCMS-EGFP expression vector, was provided by Dr Y. Aizawa (Niigata University, Niigata, Japan). Full-length cDNA encoding human *CACNA1C*, subcloned into a pcDNA expression vector, and cDNAs encoding *CACNB2b* and *CACNA2D1*, both cloned into pcDNA3.1 expression vectors, were kindly donated by Dr. C. Antzelevitch (Lankenau Institute for Medical Research, PA, United States). Full-length cDNA encoding human *SCN5A*, subcloned into a pcDNA3.1 expression vector, and full-length cDNA encoding the human sodium channel *β*1 subunit (h*β*1), subcloned into a pIRES-EGFP expression vector, were kindly donated by Dr. T. Makiyama (Kyoto University Graduate School of Medicine, Kyoto, Japan).

### 2.2 Cell preparation and transfection

The CHO cells used in the present studies were kindly provided by Dr.H.Matsuura (Department of Physiology, Shiga University of Medical Science, Otsu, Japan). The studies involving CHO cells were reviewed and approved by the Institutional Ethics Committees of both Medical School of Medicine at Xi’an Peihua University and Shiga University of Medical Science, and conducted in accordance with their guidelines. CHO cells were maintained in Dulbecco’s modified Eagle’s medium/Ham’s F-12 (DMEM/F-12) supplemented with 10% fetal bovine serum and antibiotics (100 IU mL^−1^ penicillin and 100 μg mL^−1^ streptomycin) under a humidified atmosphere of 5% CO_2_/95% air at 37 °C. Cells were passaged twice weekly by harvesting with trypsin-EDTA, and subsets of treated cells were seeded onto glass coverslips (5 × 3 mm^2^) for transfection.

2–3 days after transfection, cells attached to a glass coverslip were transferred to a 0.5-ml bath chamber perfused with external bath solution at a flow rate of 2 mL/min and maintained at 25 °C (for IKs, IKr and Ito), 22–23 °C (for INa), and 37 °C (for IK1 and ICa-L). To transiently express the macromolecular channel complexes mediating *I*_Ks_, *I*_Kr_, *I*_to_, *I*_K1_, *I*_Na_, or *I*_Ca-L_, CHO cells were transfected with the following plasmid combinations: *KCNQ1* + *KCNE1* (0.5 + 0.5 µg), *KCNH2* (hERG) + GFP (1.0 + 0.5 µg), Kv4.3 (*KCND3*) + *KCNIP2b* + *KCNE2* (1.0 + 1.0 + 1.0 µg), *KCNJ2* (1.0 µg), *SCN5A*+ h*β*_1_ (1.0 + 1.0 µg), or *CACNA1C* + *CACNB2b* + *CACNA2D1* + GFP (1.0 + 1.0 + 1.0 + 0.25 µg). All transient transfections were performed using Lipofectamine (Invitrogen; Thermo Fisher Scientific, Inc., Waltham, MA, United States) according to the manufacturer’s instructions.

### 2.3 Electrophysiological recordings, solutions, and data analysis

The inhibitory effects of LY294002 on cardiac currents (*I*_Ks_, *I*_Kr_ *, I*_to_, *I*_K1_, *I*_Na_, and *I*_Ca-L_) were recorded using the whole-cell patch-clamp technique on GFP-positive cells 2–3 days post-transfection. The specific voltage-clamp depolarization test protocols are illustrated as insets within each respective figure. To determine concentration–response curves, the percentage of current inhibition by LY294002 was fitted using a least-squares method to the following Hill equation:

$$\%\text{Control}=1/\left(1+\left(\frac{\text{IC}50}{\left[D\right]}\right)\right)n_{H}$$

where %Control represents the steady-state current amplitude in the presence of the drug normalized to the baseline control value (expressed as a percentage); IC_50_ is the concentration of LY294002 yielding half-maximal inhibition; *n*_H_ is the Hill coefficient; and [D] denotes the drug concentration.

Cells were continuously perfused at a flow rate of 2 mL min^−1^. The external bath solutions were compositionally structured as follows (in mM): (1) For potassium (*I*_Ks_, *I*_Kr_ *, I*_to_, *I*_K1_) and sodium (*I*_Na_) currents: 140 NaCl, 5.4 KCl, 1.8 CaCl_2_, 0.5 MgCl_2_, 0.33 NaH_2_PO_4_, 5.5 glucose, and 5.0 HEPES (pH adjusted to 7.40 with NaOH); (2) For L-type calcium (*I*_Ca-L_) currents: 130 N-methyl-D-glucamine, 5.0 KCl, 15 CaCl_2_, 1.0 MgCl_2_, and 10 HEPES (pH adjusted to 7.35 with HCl). Internal pipette solutions consisted of (in mM): (1) For potassium currents: 70 potassium aspartate, 40 KCl, 10 KH_2_PO_4_, 1.0 MgSO_4_, 3.0 Na_2_-ATP, 0.1 Li_2_-GTP, 5.0 EGTA, and 5.0 HEPES (pH adjusted to 7.20 with KOH); (2) For *I*_Na_ currents: 10 NaF, 110 CsF, 20 CsCl, 10 EGTA, and 10 HEPES (pH adjusted to 7.35 with CsOH); (3) For *I*_Ca-L_ currents: 120 CsCl, 2.0 MgCl_2_, 2.0 MgATP, 5.0 CaCl_2_, 10 EGTA, and 10 HEPES (pH adjusted to 7.25 with CsOH). Whole-cell membrane currents were recorded with an EPC-8 patch-clamp amplifier (HEKA, Lambrecht, Germany) and a resistance of 2.5 - 3.5 MΩ (IKs, IKr, Ito, ICa-L and IK1) or 1.5 - 2.0 MΩ (INa) electrodes when filled with the internal pipette solutions. All data were low-pass filtered at 1 kHz, acquired at 5 kHz through an LIH-1600 analogue-to-digital converter (HEKA) and stored on a hard disc drive by using Pulse/PulseFit software (HEKA).

LY294002 (Cat. No. 440202, CAS 154447–36-6; Calbiochem, San Diego, CA, United States) was dissolved in dimethyl sulphoxide (DMSO; Sigma) to yield stock solutions of 50 mmol L^−1^. The concentration of DMSO in the final solution was <0.1% (v/v), which had no effect on currents in the present study. In each experiment, cells were exposed to increasing concentrations of LY294002 in a cumulative manner after the inhibition elicited by the previous concentration reached a steady state.

### 2.4 Homology modeling of tetrameric Kv1.5

Human Kv1.5 channels function as homotetramers embedded in the plasma membrane, with four identical subunits enclosing a central ion-conducting pore. To generate a three-dimensional structural model of the full tetramer for molecular docking, a homology model of human Kv1.5 (KCNA5, UniProt ID: P22460) was constructed. The crystal structure of the open-state Kv1.2–Kv2.1 chimera channel (PDB ID: 2R9R; resolution: 2.4 Å) was selected as the structural template (Long et al., 2007), providing a transmembrane framework of the open voltage-gated potassium channel tetramer. Sequence alignment was performed between residues 269–526 of human Kv1.5 and residues 160–417 of 2R9R. Homology modeling was executed using SWISS-MODEL, with the 2R9R tetrameric architecture used as a global template to preserve the correct four-fold rotational symmetry. The stereochemical quality and geometry of the resulting homotetramer were evaluated using PROCHECK, confirming acceptable Ramachandran plot distribution (φ/ψ angles), bond lengths, and bond angles.

### 2.5 Molecular docking of LY294002 into the tetrameric Kv1.5 pore

The validated tetrameric Kv1.5 model was utilized for molecular docking. The two-dimensional structure of LY294002 was sketched in ChemDraw and converted to three-dimensional coordinates. Ligand energy minimization was performed, with rotatable bonds set as flexible and Gasteiger partial charges assigned. Based on established literature regarding Kv1.5 pore block (Marzian et al., 2013; Camargo-Ayala et al., 2025), the search grid box was centered on the central pore cavity lined by key residues Thr480, Val505, Ile508, and Val512. Using PyMOL measurement, the grid center was placed at coordinates (−7, 9, 14) with grid box dimensions of 35 Å × 32 Å × 21 Å, covering the central pore cavity. Semi-flexible docking was executed using AutoDock Vina. Output binding poses were ranked by binding affinity scores, and the top-ranked pose displaying a plausible binding mode was selected. PyMOL was used for visual analysis to characterize hydrogen bonds (H-bond), hydrophobic interactions, and measure interaction distances with pore-lining residues.

### 2.6 Statistical analysis

All averaged data are presented as mean ± SEM, with the number of independent experiments (n) indicated in parentheses. Statistical comparisons were performed using either Student’s *t*-test or one-way ANOVA with Dunnett’s *post hoc* test, as appropriate. Differences were considered statistically significant at *P* < 0.05.

## 3 Results

### 3.1 Inhibitory effect of LY294002 on *I*_Ks_

**FIGURE 2:**
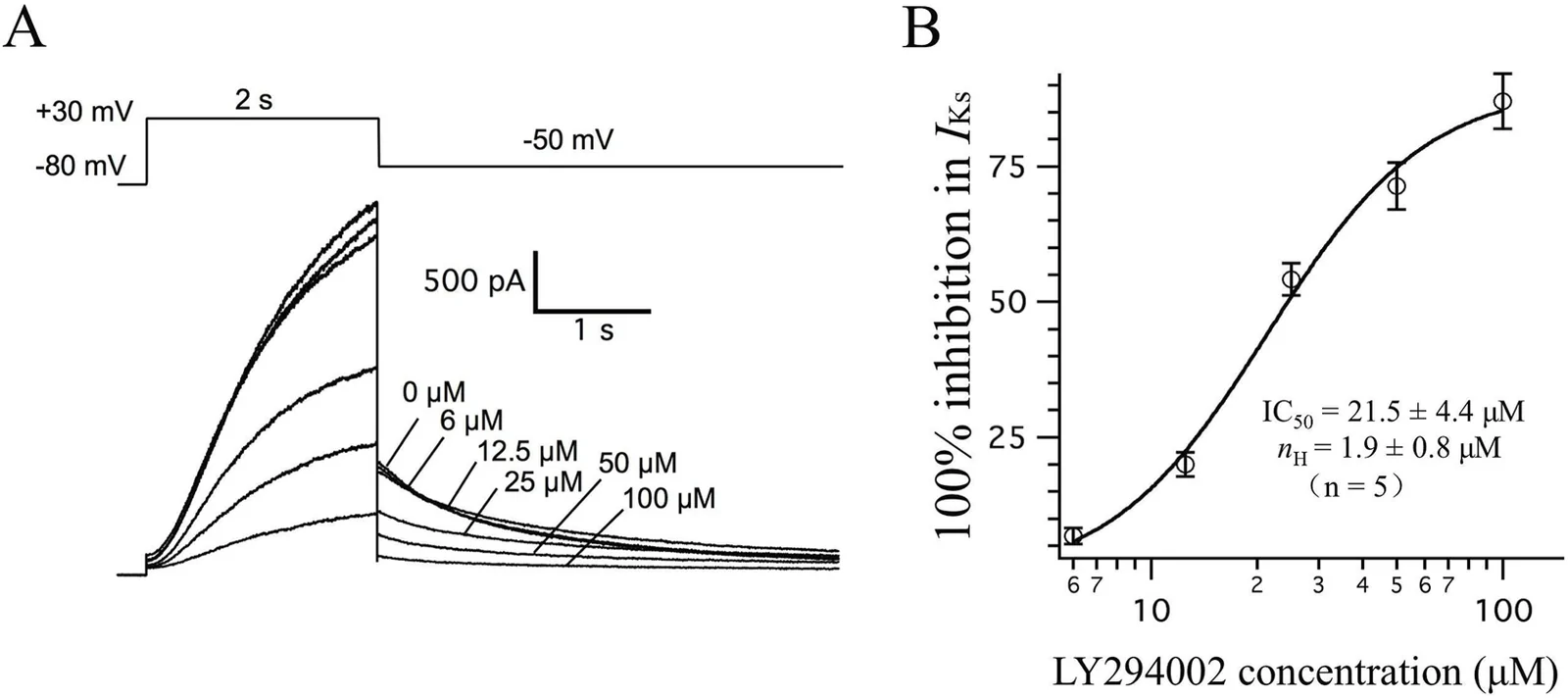
The inhibitory effect of LY294002 on *I*_Ks_ currents in CHO cells. **(A)** Superimposed *I*_Ks_ current traces elicited on repolarization to −50 mV following 2 s depolarization from a holding potential −80 mV to +30 mV in the presence of various LY294002 concentrations (6, 12.5, 25, 50 and 100 µM) in a cumulative manner. **(B)** The concentration-response relation for the LY294002 inhibition on *I*_Ks_. The solid line represents the fit of the data to the Hill equation, with an IC_50_ and *n*_H_ shown in the inset.

The slow delayed-rectifier potassium current (*I*_Ks_) is one of the main repolarizing currents expressed in atria and ventricles. To evaluate whether LY294002 exerts an inhibitory effect on *I*_Ks_, membrane currents were recorded before and during LY294002 exposure in CHO cells heterologously expressing KCNQ1/KCNE1 channel complexes (Figure 2A). *I*_Ks_ amplitude was quantified by measuring the peak tail current elicited upon repolarization to −50 mV following a 2-s depolarization to +30 mV (inset). Superimposed traces show concentration-dependent inhibition of tail currents by LY294002. Figure 2B illustrates the concentration–response curve for LY294002-mediated inhibition of *I*_Ks_; the averaged data were fitted with the Hill equation, yielding a half-maximal inhibitory concentration (IC_50_) of 21.5 μM and a Hill coefficient (*n*_H_) of 1.9. Compared with the reported IC_50_ of LY294002 for ultrarapid delayed-rectifier potassium current (*I*_Kur_) block (7.9 μM) (Wu, et al., 2009), the IC_50_ for *I*_Ks_ is approximately 2.7-fold higher.

### 3.2 Inhibitory effect of LY294002 on *I*_Kr_

**FIGURE 3:**
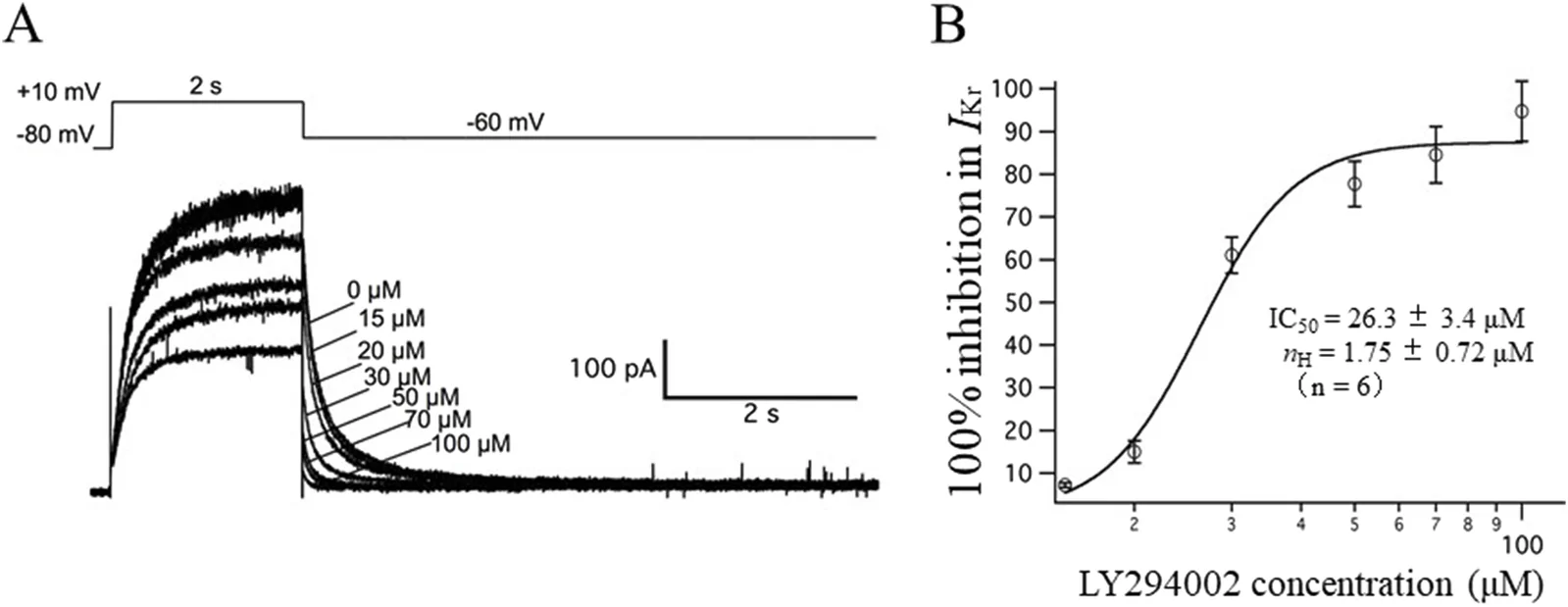
The inhibitory effect of LY294002 on *I*_Kr_ currents in CHO cells. **(A)** Superimposed *I*_Kr_ current traces elicited by applying 2 s depolarizing pulses from a holding potential of −80 mV to +10 mV and then repolarization to −60 mV, in the presence of various LY294002 concentrations (15, 20, 30, 50, 70 and 100 µM). **(B)** The concentration-response relation for the LY294002 inhibition on *I*_Kr_. The solid line represents the fit of the data to the Hill equation, with an IC_50_ and *n*_H_ shown in the inset.

The rapid delayed-rectifier potassium current (*I*_Kr_) is another major repolarizing current expressed in atria and ventricles. Owing to its unique pore architecture (spacious inner cavity and aromatic drug-binding sites in the S6 domain), *I*_Kr_ displays unusual susceptibility to structurally diverse compounds (Mitcheson et al., 2000). To assess the inhibitory effect of LY294002 on *I*_Kr_, membrane currents were recorded at LY294002 concentrations of 15–100 µM in CHO cells expressing hERG (Figure 3A). *I*_Kr_ was elicited using the voltage-clamp protocol shown in the inset, and amplitude was quantified from tail currents. Superimposed traces show concentration-dependent inhibition of tail currents by LY294002. Figure 3B illustrates the concentration–response curve for LY294002-mediated inhibition of *I*_Kr_; fitting the mean data yielded an IC_50_ of 26.3 μM and an *n*_H_ of 1.75. This IC_50_ is approximately 3.3-fold higher than that reported for LY294002 inhibition of *I*_Kur_.

### 3.3 Inhibitory effect of LY294002 on *I*_to_

**FIGURE 4:**
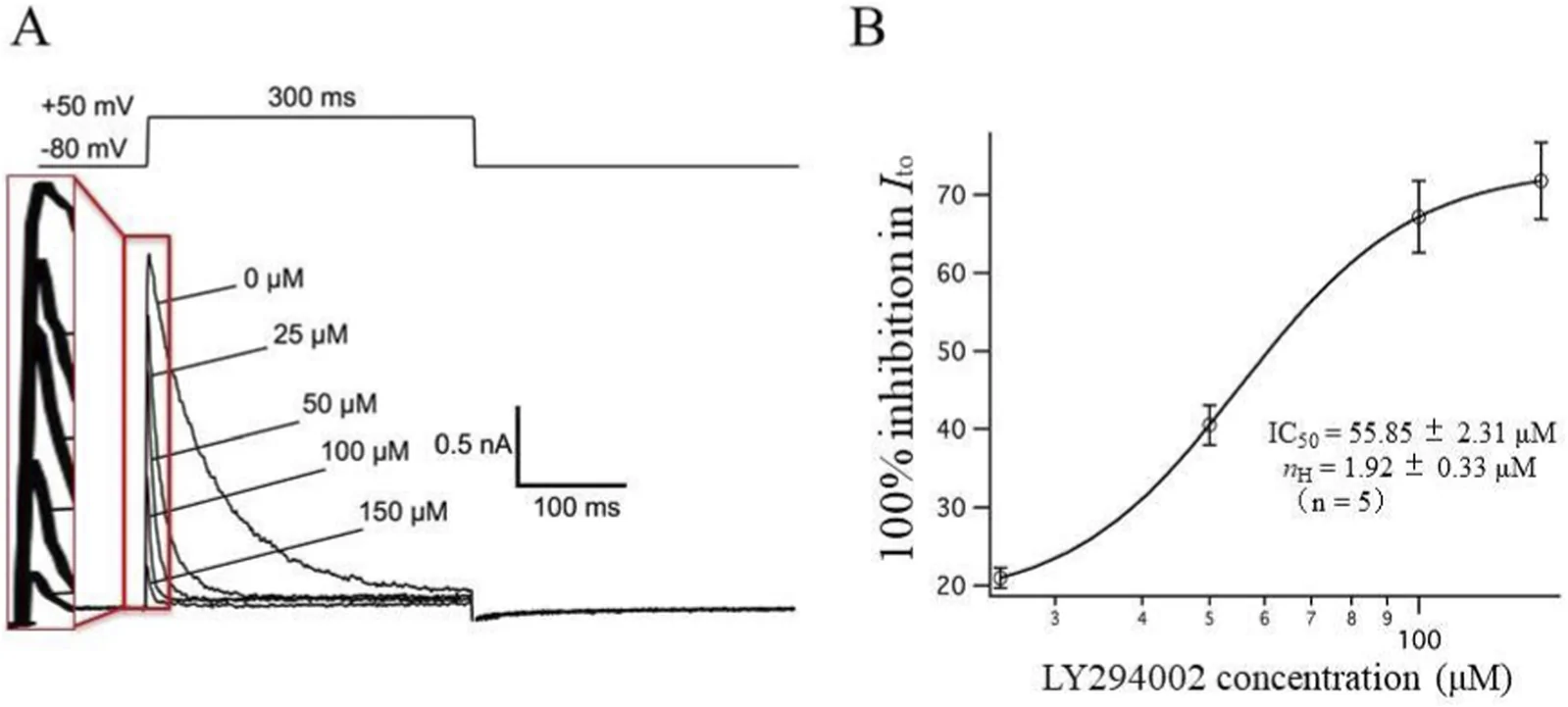
The inhibitory effect of LY294002 on *I*_to_ currents in CHO cells. **(A)** Superimposed *I*_to_ current traces elicited by applying 300 ms depolarizing pulses from a holding potential of −80 mV to +50 mV in the presence of various LY294002 concentrations (25, 50, 100 and 150 µM). **(B)** Concentration-response relation for the inhibition of *I*_to_ peak currents by LY294002. The solid line represents the fit of the data to the Hill equation, with an IC_50_ and *n*_H_ shown in the inset.

The transient outward potassium current (*I*_to_) is critical for regulating myocardial electrical properties during the initial depolarization and early repolarization phases of the cardiac action potential (AP) and has been implicated in drug-induced arrhythmias (Ni et al., 2019; Cubeddu, 2016). To determine whether LY294002 inhibits *I*_to_, membrane currents were recorded in CHO cells expressing Kv4.3 (KCND3), KCNIP2b, and KCNE2 (Figure 4A). Blockade of *I*_to_ by LY294002 was observed at concentrations of 25–150 µM. Superimposed traces, elicited by the voltage-clamp protocol shown in the inset, demonstrate concentration-dependent inhibition of peak currents. Figure 4B illustrates the corresponding concentration–response curve; the mean data were well fitted with a Hill equation, yielding an IC_50_ of 55.8 μM and an *n*_H_ of 1.92.

### 3.4 Inhibitory effect of LY294002 on *I*_K1_

**FIGURE 5:**
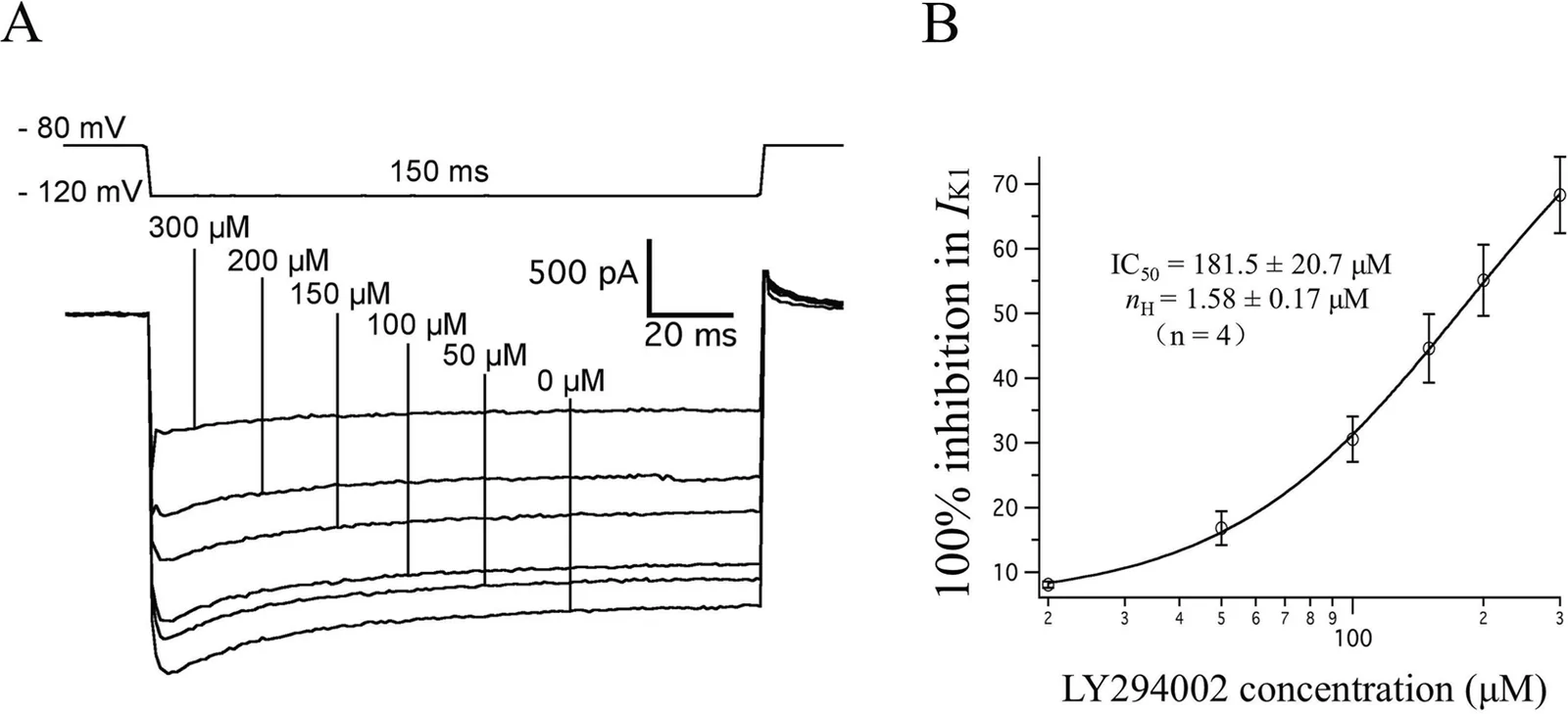
The inhibitory effect of LY294002 on *I*_K1_ currents in CHO cells. **(A)** Superimposed *I*_K1_ current traces evoked by applying a 150 ms square pulse from a holding potential of −80 mV to −120 mV in the presence of LY294002 at various concentrations (50, 100, 150, 200 and 300 µM). **(B)** Concentration-response relation for the inhibition of *I*_K1_ currents by LY294002. The solid line represents the fit of the data to the Hill equation, with an IC_50_ and *n*_H_ shown in the inset.

The inward rectifier potassium current (*I*_K1_) plays a crucial role in stabilizing the resting membrane potential and shaping the terminal repolarization phase of the cardiac AP. To evaluate the effect of LY294002 on *I*_K1_, membrane currents were recorded in CHO cells transfected with *KCNJ2* and exposed to 50–300 µM LY294002. Figure 5A depicts superimposed current traces evoked by 150-ms square pulses applied from a holding potential of −80 mV to a test potential of −120 mV, demonstrating concentration-dependent inhibition on *I*_K1_ by LY294002. Figure 5B illustrates the corresponding concentration–response curve; the mean data were reasonably fitted with the Hill equation, with an IC_50_ of 181.5 μM and an *n*_H_ of 1.58.

### 3.5 Inhibitory effect of LY294002 on *I*_Na_

**FIGURE 6:**
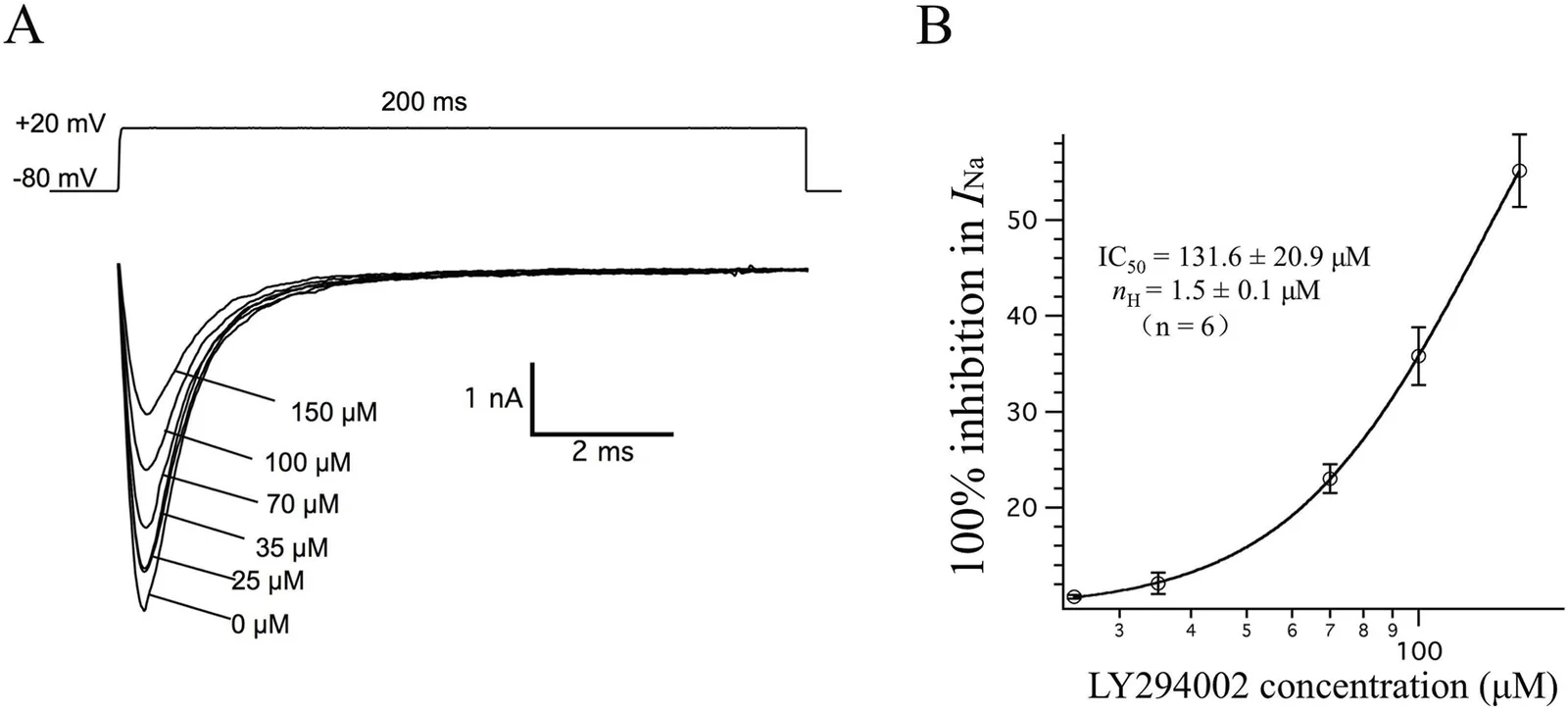
The inhibitory effect of LY294002 on *I*_Na_ currents in CHO cells. **(A)** Superimposed *I*_Na_ current traces elicited by applying 200 ms depolarizing pulses from a holding potential of −80 mV to +20 mV in the presence of various LY294002 concentrations (25, 35, 70, 100 and 150 µM). **(B)** Concentration-response relation for the inhibition of *I*_Na_ currents by LY294002. The solid line represents the fit of the data to the Hill equation, with an IC_50_ and *n*_H_ shown in the inset.

The voltage-gated sodium current (*I*_Na_) underlies the initial upstroke and propagation of the AP in cardiac myocytes, thereby determining cardiac excitability and the conduction velocity of electrical stimuli through the heart. Dysregulation of *I*_Na_ is responsible for several types of arrhythmias, including drug-induced variants (Tisdale et al., 2020; Amin et al., 2010). To assess the inhibitory effect of LY294002 on *I*_Na_, membrane currents were recorded in CHO cells co-transfected with *SCN5A* and h*β*_1_ and exposed to 25–150 µM LY294002 (Figure 6A). Superimposed current traces, evoked by the protocol shown in the inset, indicate concentration-dependent inhibition of *I*_Na_ by LY294002. Figure 6B illustrates the corresponding concentration–response curve, yielding an IC_50_ of 131.6 μM and an *n*_H_ of 1.5.

### 3.6 Inhibitory effect of LY294002 on *I*_Ca-L_

**FIGURE 7:**
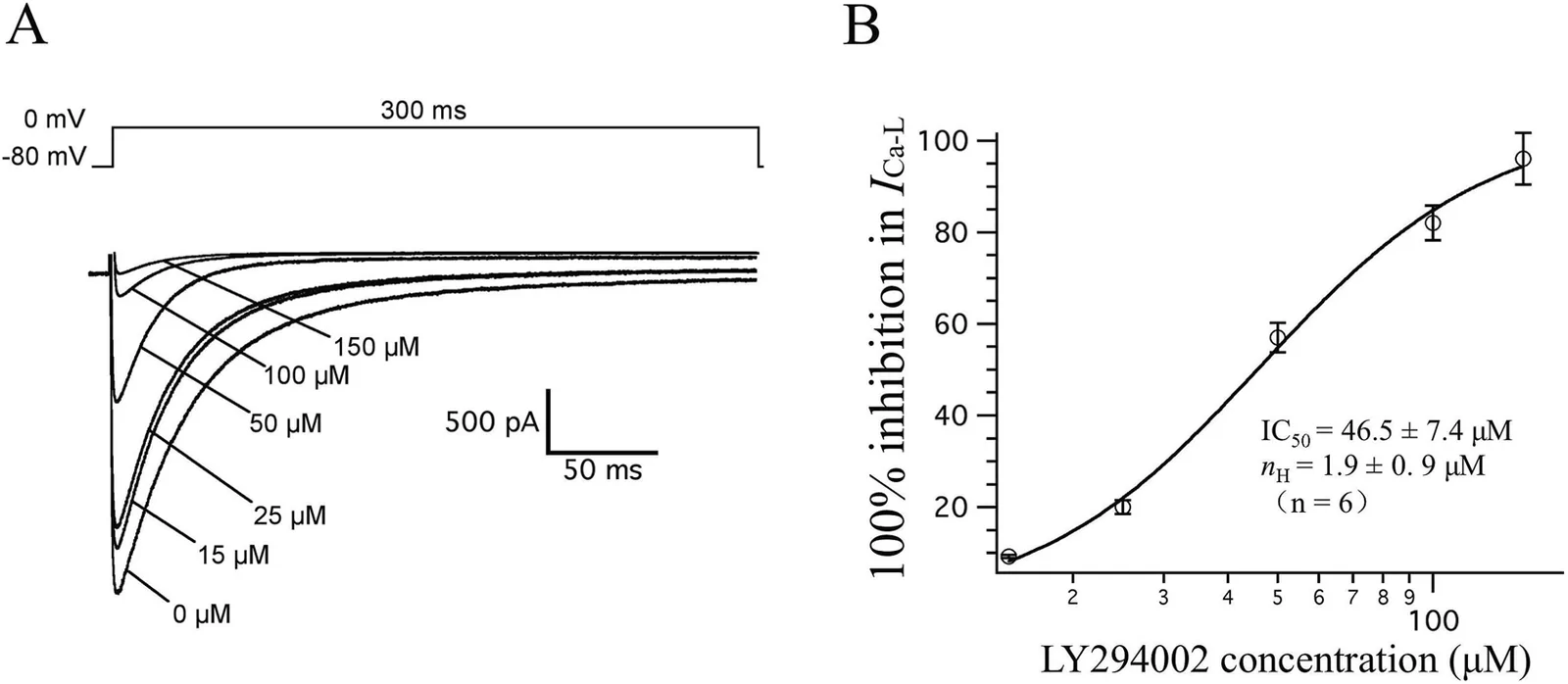
The inhibitory effect of LY294002 on *I*_Ca-L_ currents in CHO cells. **(A)** Superimposed *I*_Ca-L_ current traces elicited by 300 ms depolarizing voltage to 0 mV from a holding potential of −80 mV in the presence of various LY294002 concentrations (15, 25, 50, 100, and 150 µM). **(B)** Concentration-response relation for the inhibition of *I*_Ca-L_ currents by LY294002. The solid line represents the fit of the data to the Hill equation, with an IC_50_ and *n*_H_ shown in the inset.

The L-type calcium current (*I*_Ca-L_) plays a critical role in cardiac AP generation, morphology, and duration, and is essential for maintaining calcium homeostasis in cardiac myocytes (Eisner et al., 2017). Dysregulation of *I*_Ca-L_ has been implicated in a wide range of cardiac diseases, including multiple forms of arrhythmias. To evaluate the effect of LY294002 on *I*_Ca-L_, membrane currents were recorded in CHO cells co-transfected with *CACNA1C*, *CACNB2b*, and *CACNA2D1* (Figure 7A). Superimposed *I*_Ca-L_ traces, elicited by the protocol shown in the inset, showed concentration-dependent inhibition across a range of 15–150 µM LY294002. Figure 7B shows the corresponding concentration–response curve; the calculated IC_50_ was 46.5 μM, and the *n*_H_ was 1.97.

### 3.7 Molecular docking of LY294002 within the tetrameric Kv1.5 pore cavity

**FIGURE 8:**
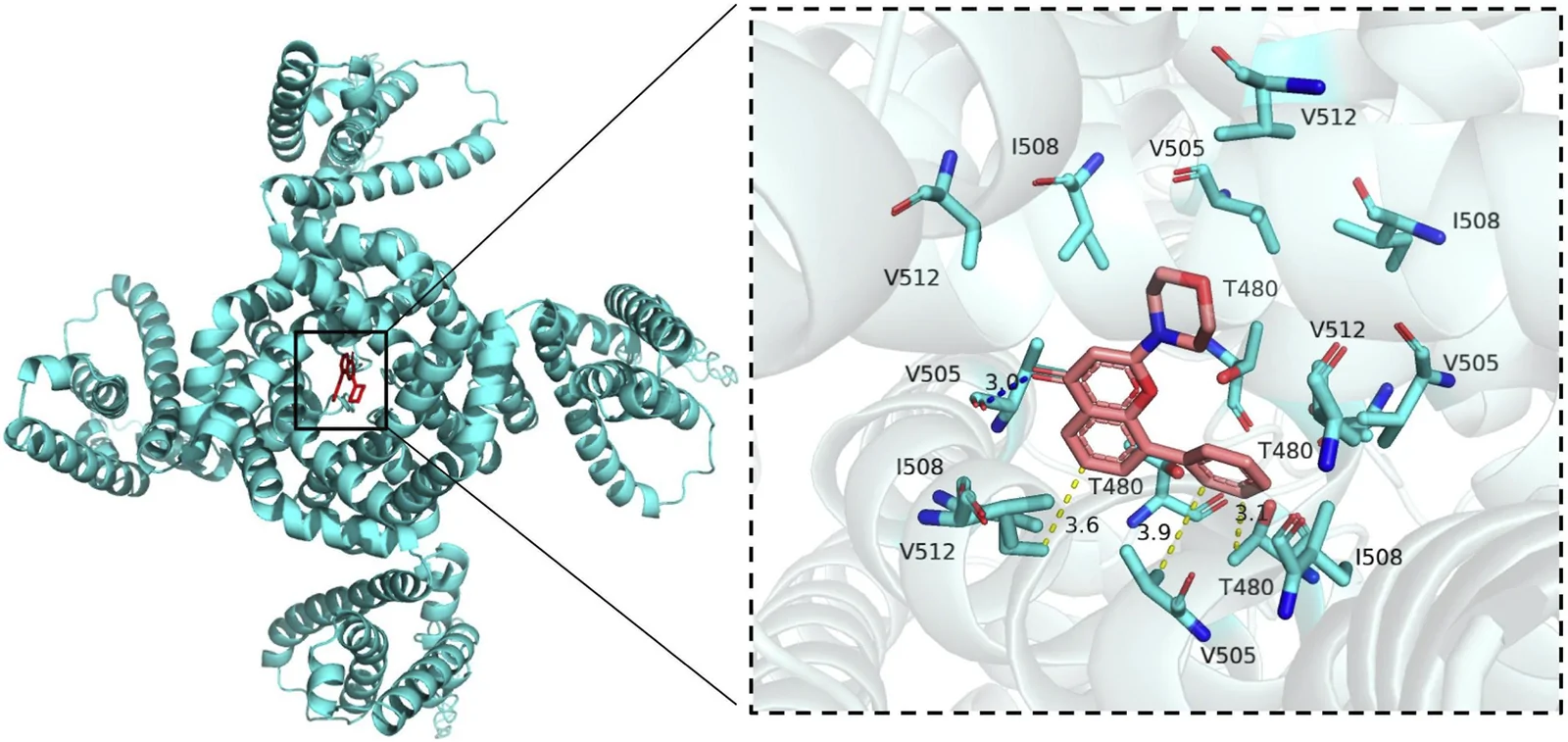
Molecular docking of LY294002 within the tetrameric Kv1.5 pore cavity.

The binding mode of LY294002 with Kv1.5 is shown in Figure 8. LY294002 is accommodated in the inner pore cavity surrounded by four subunits, with its long molecular axis oriented along the central pore axis. The predicted binding affinity of LY294002 in the central pore cavity is −8.3 kcal/mol. The backbone carbonyl oxygen of Val505 forms a H-bond with LY294002 (interaction distance: 3.0 Å). In addition, hydrophobic contacts are observed with Thr480, Val505 (from an adjacent subunit), and Ile508, with distances of 3.1 Å, 3.9 Å, and 3.6 Å, respectively. These binding sites are partially consistent with our previous patch clamp data and other reports on the binding sites of Kv1.5 inhibitors (Wu, et al., 2009; Kiper et al., 2021).

## 4 Discussion

The electrophysiological data in the present study demonstrate that LY294002 inhibits multiple cardiac ion currents (*I*_Ks_, *I*_Kr_, *I*_to_, *I*_K1_, *I*_Ca-L_, and *I*_Na_) in a dose-dependent manner, yet its potency is consistently lower than that required to suppress *I*_Kur_. Notably, the IC_50_ values for *I*_K1_ and *I*_Na_ were much higher than that for *I*_Kur_ (22.9-fold and 16.6-fold, respectively), whereas those for *I*_Ks_ and *I*_Kr_ were only ∼3-fold higher, underscoring a relative selectivity for Kv1.5/*I*_Kur_. Molecular docking analysis suggested that LY294002 preferentially antagonizes *I*_Kur_ by precisely occupying key gating domains unique to the channel’s architecture.

Currently available antiarrhythmic drugs for the treatment of AF are not sufficiently effective and are burdened by cardiac and extracardiac side effects that may offset their therapeutic benefits (Brundel et al., 2022). Class I agents (e.g., flecainide and propafenone), which primarily target Na_v_1.5 (*I*_Na_), are limited by negative inotropic effects and ventricular proarrhythmic risks, particularly in patients with structural heart disease and severe left ventricular dysfunction. Class III drugs (e.g., dofetilide and sotalol), which primarily inhibit Kv11.1 (*I*_Kr_), as well as the multi-channel blocker amiodarone, are also compromised by their serious cardiac and extracardiac side effects (Heijman et al., 2021). Therefore, recent research efforts have shifted toward developing atrial-selective potassium channel inhibitors, including agents targeting the atrial-specific Kv1.5 (*I*_Kur_) channel (Al-Owais et al., 2023). To date, however, only vernakalant (Brinavess) has been introduced into clinical therapeutics for AF in Europe as an *I*_Kur_ inhibitor. Although vernakalant exhibits favorable efficacy in restoring sinus rhythm and reducing AF recurrence, with a superior safety profile, its most important antiarrhythmic action is considered to be blockade of the sodium current (*I*_Na_) (Borrego et al., 2021). Our previous study demonstrated that LY294002 effectively blocked hKv1.5 channel conductance in CHO cells, with a remarkably low IC_50_ (7.9 μM), showing concentration-, time-, and frequency-dependent inhibition (Wu, et al., 2009). These findings prompted us to investigate whether the compound exerts a selective inhibitory action on other cardiac currents.

Compared with vernakalant (Fedida et al., 2005), LY294002 exhibits stronger inhibition of *I*_Kur_ but weaker activity against *I*_Ks_, *I*_Kr_, *I*_to_, *I*_K1_, *I*_Ca-L_, and *I*_Na_, highlighting a distinctive pharmacological profile. These findings suggest that LY294002 may serve as a useful agent for atrial-selective repolarization and could hold potential as a lead compound for therapeutic strategies in AF and other conditions associated with Kv1.5 dysfunction, including cancer (Comes et al., 2013) and hypoxic pulmonary vasoconstriction (Archer et al., 2001).

In human cardiomyocytes, *I*_Na_ and *I*_Ca-L_ contribute to the upstroke and plateau phases of the cardiac AP, respectively, whereas *I*_Ca-L_ also underlies the pacemaker potential in sinoatrial cells. During the plateau phase, repolarization is governed by the combined actions of *I*_Ca-L_ and potassium currents including *I*_Ks_, *I*_Kr_, and *I*_to_. While *I*_K1_ plays an important role in stabilizing the resting membrane potential and shaping the initial depolarization as well as final repolarization of the cardiac AP. Collectively, these channels govern key cardiac properties such as excitability, AP duration, and refractoriness (Varró et al., 2021). Drug-induced abnormalities in these currents are strongly associated with clinical rhythm disorders such as torsades de pointes (TdP), AF, atrioventricular nodal reentrant tachycardia, and Brugada syndrome (Tisdale et al., 2020). The markedly lower inhibitory concentration of LY294002 for *I*_Kur_ compared with other major cardiac ion currents indicates a selective blockade of Kv1.5, with minimal off-target effects. Although this selective profile is particularly advantageous for AF therapy and may extend to other conditions associated with Kv1.5 dysfunction. If LY294002 can be structurally modified, it is believed that a more ideal specific *I*_Kur_ blocker can be obtained.

The *I*_Kur_ is a major repolarizing current in human atrium and a potential target for treating atrial arrhythmias. Selective blockade of *I*_Kur_ by LY294002 may be beneficial to prolong AP duration and atrial refractory period in chronic AF than in sinus rhythm (SR), and may therefore be more efficient in terminating AF than in maintaining SR (Wettwer et al., 2004; Ellinwood et al., 2017). Although there is significant controversy over the exact anti-AF effects of some preclinical trials of Kv1.5 blockers such as BMS-394136, MK-0448 and XEN-D0103 (Wiedmann and Schmidt, 2024). However, in the last few years, some studies have begun to delve deeper into the search for higher affinity and selective blockade of Kv1.5 channels, such as hydrogen sulfides (Al-Owais et al., 2023), peptides (Borrego et al., 2021), Lgi3-4 proteins (Socuellamos et al., 2026), and synaptosomal associated protein 25 kDa (Su et al., 2024). These chemicals may potentially be of pathophysiological significance in regulating atrial function and supports a putative selective therapeutic role for Kv1.5 inhibition in AF.

MD prediction indicates that LY294002 is located in the central pore of Kv1.5, with a binding affinity of −8.3 kcal/mol. The ligand forms hydrogen bonds and hydrophobic interactions with pore-lining residues. The predicted binding mode is consistent with canonical pore-blocking sites described in previous literature (Kiper et al., 2021). These data suggest that LY294002 may inhibit Kv1.5 primarily through spatial occupancy within the central cavity, physically obstructing K^+^ ion permeation.

In conclusion, patch-clamp experiments demonstrate that the inhibitory concentration of LY294002 on other major cardiac ion currents is significantly higher than that for *I*_Kur_. Concurrently, molecular docking analysis suggests that LY294002 exerts its inhibitory effect on *I*_Kur_ by physically occupying key gating regions within the Kv1.5 channel.

### 4.1 Limitations

This study was conducted using heterologous expression systems, which may not completely recapitulate the physiological environment or complex regulatory pathways of native human cardiac ion channels. Furthermore, the drug concentration used to measure the IC_50_ of LY294002 on ion channels may not be reasonable enough, resulting in differences between the measured and actual IC_50_ values. Finally, molecular docking calculations in the present study were constrained by the accuracy of the homology model, sequence divergence from the template (Kv1.2–Kv2.1 chimera), and the absence of a lipid bilayer environment or dynamic channel gating fluctuations. The docking data should be interpreted as a complementary structural hypothesis to our electrophysiological findings.

## Data Availability

The original contributions presented in the study are included in the article/supplementary material, further inquiries can be directed to the corresponding author.
